# Diabetestechnologie (Update 2026)

**DOI:** 10.1007/s00508-025-02630-7

**Published:** 2026-04-30

**Authors:** Ingrid Schütz-Fuhrmann, B. Rami-Merhar, Sabine E. Hofer, Elke Fröhlich-Reiterer, Martin Tauschmann, Julia K. Mader, Michael Resl, Alexandra Kautzky-Willer, Yvonne Winhofer-Stöckl, Thomas Wascher, Jürgen Harreiter, Lars Stechemesser, Sandra Zlamal-Fortunat, Raimund Weitgasser

**Affiliations:** 1 Medizinische Abteilung mit Stoffwechselerkrankungen und Nephrologie, Karl Landsteiner Institut für Endokrinologie und Stoffwechselerkrankungen, Klinik Hietzing, Wien, Österreich; 2 Universitätsklinik für Kinder- und Jugendheilkunde, Medizinische Universität Wien, Wien, Österreich; 3 Universitätsklinik für Pädiatrie 1, Medizinische Universität Innsbruck, Innsbruck, Österreich; 4 Universitätsklinik für Kinder- und Jugendheilkunde, Klinische Abteilung für Allgemeine Pädiatrie, Medizinische Universität Graz, Graz, Österreich; 5 Universitätsklinik für Kinder- und Jugendheilkunde, Klinische Abteilung für Pädiatrische Pulmologie, Allergologie und Endokrinologie, Medizinische Universität Wien, Wien, Österreich; 6 Klinische Abteilung für Endokrinologie und Diabetologie, Universitätsklinik für Innere Medizin, Medizinische Universität Graz, Graz, Österreich; 7 Abteilung für Innere Medizin I, Krankenhaus Barmherzige Brüder Linz, Linz, Österreich; 8 Klinische Abteilung für Endokrinologie und Stoffwechsel, Universitätsklinik für Innere Medizin III, Medizinische Universität Wien, Wien, Österreich; 9 1. Medizinische Abteilung, Hanusch-Krankenhaus, Wien, Österreich; 10 Abteilung für Innere Medizin mit Palliative Care, Landesklinikum Scheibbs, Niederösterreich, Österreich; 11 Abteilung für Endokrinologie und Stoffwechsel, Universitätsklinik für Innere Medizin III, Medizinische Universität Wien, Wien, Österreich; 12 Universitätsklinik für Innere Medizin I mit Gastroenterologie-Hepatologie, Nephrologie, Diabetologie und Stoffwechselerkrankungen, Medizinische Universität Wien, Wien, Österreich; 13 Universitätsklinikum, Paracelsus Medizinische Privatuniversität, Salzburg, Österreich; 14 Abteilung für Innere Medizin und Gastroenterologie, Hepatologie, Endokrinologie, Rheumatologie und Nephrologie, Klinikum Klagenfurt, Klagenfurt, Österreich; 15 Kompetenzzentrum Diabetes, Mavie Med Privatklinik Wehrle Diakonissen, Salzburg, Österreich

**Keywords:** Insulinpumpentherapie, CGM/kontinuierliche Glukosemessung, AID/hybrid closed loop, Handy-Apps, Telemedizin, Insulin pump therapy, Continuous glucose monitoring, Automated insulin delivery systems, Mobile telephone apps, Telemedicine

## Abstract

Diese Leitlinie repräsentiert die Empfehlungen der Österreichischen Diabetes Gesellschaft (ÖDG) zur Nutzung von Diabetes-Technologie (kontinuierliche Glukosemesssysteme [CGM], Insulinpumpentherapie, Automated-Insulin-Delivery-Systeme [AID], „connected insulin pens“, Diabetes-Apps) und den Zugang zu diesen technologischen Innovationen für Menschen mit Diabetes mellitus. Die Leitlinie wurde basierend auf aktueller wissenschaftlicher Evidenz erstellt.

## Abstract (en)

These guidelines represent the recommendations of the Austrian Diabetes Society (ÖDG) on the use of diabetes technology (continuous glucose monitoring, CGM; insulin pump therapy; automated insulin delivery systems, AID, connected insulin pens, diabetes apps) and the access to these technological innovations for people with diabetes mellitus. The guidelines were based on current scientific evidence.

## Insulinpumpen und kontinuierliche Glukosemessung (CGM)

Neben der Entwicklung von neuen Insulinen mit vorteilhaften Wirkprofilen und innovativen Medikamenten haben Menschen mit Diabetes mellitus über die letzten Jahrzehnte v. a. auch von Fortschritten und Innovationen im Bereich der Diabetestechnologie profitiert. Insulinpumpen zur kontinuierlichen Insulinabgabe und Sensoren zur kontinuierlichen Glukosemessung haben sich sowohl im pädiatrischen Bereich als auch in der Betreuung von Erwachsenen mit Typ-1-Diabetes (T1D) durchgesetzt. Im Vergleich zur Basis-Bolus-Therapie mittels Insulinpens und kapillären Blutglukosemessungen sind die Verwendung von Glukosesensoren und die Verwendung von Insulinpumpen und automatischen Insulinabgabesystemen mit einer Reduktion der HbA_1c_-Werte sowie einer Verbesserung der Metriken, gemessen mittels CGM, und damit Verbesserung der metabolischen Kontrolle assoziiert [1–7].

## Therapieziele werden auf Technologie ausgerichtet

**Abb. 1:**
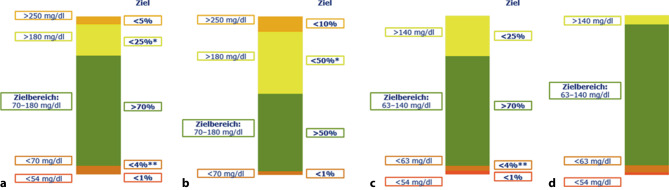
Zeit im Zielbereich für einzelne Patient:innengruppen. *(Sternchen)* Beinhaltet Prozent der Werte > 250 mg/dl; *(2* *Sternchen) *beinhaltet Prozent der Werte < 54 mg/dl; ^†^ bei Personen < 25 Jahre, falls das HbA_1c_-Ziel bei 7,5 % (58 mmol/mol) liegt, dann sollte die TIR bei ~ 60 % gelegen sein; ‡ Prozent der Zeiten in den einzelnen Bereichen basieren auf geringer Datenlage; ^§^ keine Prozentangaben möglich aufgrund zu geringer Datenlage; es wird angenommen, dass so viel Zeit wie möglich im Zielbereich mit möglichst wenig Zeit über dem Zielbereich angestrebt werden soll. **a** T1D und T2D, **b** ältere Menschen/Menschen mit hohen Risikofaktoren mit T1D/T2D, **c** Schwangerschaft: T1D, **d** Schwangerschaft: Gestationsdiabetes/T2D. *GDM* Gestationsdiabetes, *T1D* Typ-1-Diabetes, *T2D* Typ-2-Diabetes. (Mod. nach Battelino et al. [9])

Der etablierten Messgröße HbA_1c_ liegt ein Glukosemittelwert über 2 bis 3 Monate zugrunde. Der HbA_1c_-Wert gilt als gutes Maß für die durchschnittliche Glykämie, nicht jedoch für Hypoglykämien und Glukoseschwankungen. Letztere werden in Analogie zur Hyperglykämie mitursächlich für mikro- und makrovaskuläre Komplikationen gesehen [8]. Durch die immer größere Verbreitung von CGM-Systemen sowohl bei Benutzer:innen von Insulinpumpen als auch Insulinpens stehen deutlich mehr Daten zur Verfügung als bei der herkömmlichen kapillären Blutglukosemessung. Der Begriff „time in range“ (TIR = Zeit im Zielbereich), d. h. der Prozentsatz an Zeit mit Sensorglukosewerten im Bereich zwischen 70 und 180 mg/dl, hat in den letzten Jahren zunehmend an Bedeutung gewonnen und ist zu einer neuen Messgröße zusätzlich zum HbA_1c_ geworden [9, 10]. Mehr als 70 % der Glukosewerte eines Tages sollten beim Großteil der Menschen mit Diabetes mellitus in diesem Zielbereich verbracht werden [9]. Eine geringere TIR von 50 % ist bei älteren Personen oder Personen mit hohem Risiko als Ziel anzustreben. Ein engerer Zielbereich von 63–140 mg/dl ist in der Schwangerschaft („time in range pregnancy“ = TIR_p_) empfohlen (s. auch Abb. 1). Auch abseits der Gravidität streben Menschen mit Diabetes und ihre Behandlungsteams immer mehr Normoglykämie an, weswegen rezent die sog. „time in normal glycemia (TiNG)“ – also der Anteil der Werte im normalen Bereich von 70–140 mg/dl – eingeführt wurde und als mögliches Therapieziel diskutiert wird [11].

**Abb. 2:**
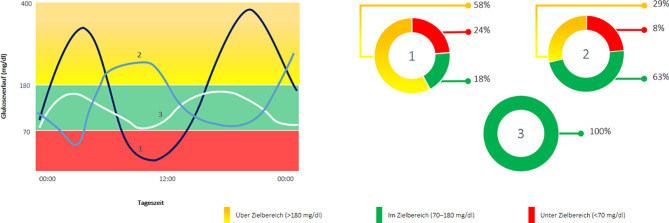
Ein definierter HbA_1c_ kann mit ganz unterschiedlichen Glukoseverläufen einhergehen. Das CGM zeigt die glykämische Variabilität bei Personen mit Diabetes mellitus und demselben HbA_1c_. Der Einsatz von CGM ermöglicht eine Quantifizierung der TAR, TIR und TBR, um individuelle klinische Entscheidungen zu treffen

Eine TIR von 70 % korreliert dabei mit einem HbA_1c_-Wert von 7,0 % (53 mmol/mol) oder knapp darunter [9]. Hinsichtlich der Hypoglykämie wurde das Ziel mit weniger als 4 % des Tages unter 70 mg/dl und weniger als 1 % unter 54 mg/dl als Konsensusempfehlung formuliert (Abb. 1; [9]). Die Zeit im Zielbereich von 70 % zeigt auch eine gute Korrelation hinsichtlich des Auftretens von Folgeerkrankungen, ähnlich mit jener der HbA_1c_-Werte [12]. Zudem stellt die durchschnittliche Glukose („mean glucose“) ein Maß für die Hyperglykämie und der „glucose coefficient of variation“ (CV) ein Maß für die Glukosevariabilität und somit die Glukoseschwankungen dar. Der von Menschen mit Diabetes oft erwähnte „glucose management indicator“ (GMI) als errechneter HbA_1c_-Wert ersetzt die laborchemische Messung nicht und kann in einigen Fällen falsch sein ([13–15]; Abb. 2).

## Kontinuierliche Glukosemessung (CGM)

Unter kontinuierlicher Glukosemessung („continuous glucose monitoring“ [CGM]) versteht man die Messung der Glukose im subkutanen Fettgewebe alle 1–15 min mittels eines trans- oder subkutanen Sensors. Die Zeitverzögerung zur Blutglukose beträgt je nach Messsystem ca. 10–20 min, wird aber durch die aktuelle Sensormesstechnologie nahezu ausgeglichen, sodass Sensormessungen direkt für Therapieentscheidungen herangezogen werden können. Die Messwerte werden mittels eines Empfängergerätes erfasst und gespeichert und die Daten mithilfe spezieller Softwareprogramme ausgewertet. CGM kann am Endgerät (Receiver, Lesegerät, Smartphone) gestreamt werden. Die Glukosewerte werden gemeinsam mit einer Verlaufskurve der letzten Stunden auf einem Display angezeigt. Zusätzlichen Informationsgehalt liefern die mit Richtungspfeilen angezeigten Trends. Während bei der blutigen Glukosemessung nicht erfasst werden kann, in welche Richtung der weitere Glukoseverlauf geht, können kontinuierliche Messsysteme über die Dynamik der weiteren Glukoseverläufe informieren.

Individuell können Benachrichtigungen und Alarme eingestellt werden, die bei Über- oder Unterschreiten definierter Grenzwerte sowie vor zu raschen Glukoseanstiegen oder -abfällen warnen. Die Alarme können akustisch oder per Vibration erfolgen. Dabei kann der Alarm bereits prädiktiv je nach Einstellung und System erfolgen. Die zugehörige Computer-App ermöglicht statistische und grafische Auswertungen zum Glukoseverlauf über Tage bis Monate.

Viele Studien wurden noch mit „intermittently scanned CGM“ (isCGM) durchgeführt. Dabei erhielt die/der Anwender:in nur Wert und Trend, wenn sie/er einen Scan des Sensors mit dem Lesegerät/Smartphone durchführte. In einer fortgeschrittenen Form war eine Alarmfunktion bereits gegeben. Heute ist die Streamingfunktion obligat.

Bei Menschen mit T1D gilt CGM als Methode der Wahl für die Glukosemessung [11].

Neben der therapeutischen Anwendung von CGM wird diese Technologie von Diabetesteams genützt, um individualisierte Therapieempfehlungen anhand der Glukoseprofile und standardisierten Auswertung der Glukoseparameter zu geben.

## Stellenwert der Blutglukosemessung

Die Blutglukosemessung ist nach wie vor eine etablierte Methode, um Menschen mit Diabetes Selbstkontrolle zu ermöglichen und um dem Diabetesteam Daten für die Therapieanpassung und die Schulung zur Verfügung zu stellen. Blutglukosewerte sollen prä- und postprandial nach festem Schema erfasst werden. Häufigkeit und Zeitpunkte der Messung richten sich nach Therapieform und Hypoglykämierisiko. Bei Personen unter Insulintherapie führt eine höhere Messfrequenz zu einer besseren glykämischen Kontrolle und zu einer geringeren glykämischen Variabilität [16, 17]. Eine Metaanalyse aus 2018 stellt dar, dass in RCT bei nicht insulinpflichtigen Menschen mit T2D eine regelmäßige Blutzuckerselbstkontrolle den HbA_1c_-Wert signifikant senken konnte [17]. Dieser Effekt fällt größer aus, wenn die Kontrollen nach einem festen Schema erfolgen und die Messergebnisse aktiv zur Anpassung der Therapie genutzt werden.

## „Connected“ Insulinpens

Darunter versteht man Insulinpens, die die Insulinabgabe automatisch speichern und an Diabetes-Apps übermitteln können. Das System kann einen Rechner beinhalten, der abhängig vom aktiven Insulin, dem Kohlehydrat-Insulin-Faktor und dem Korrekturfaktor einen Bolusvorschlag erstellt. Die Beziehung zwischen Insulin, Mahlzeit und körperlicher Aktivität kann durch diese Systeme aufgezeigt werden und Einblicke in die Optimierung von Insulinregimen geben [18]. Erste Analysen dieser Systeme weisen auf einen klinischen Benefit hin und geben Einblick in das Injektionsverhalten von Menschen mit Diabetes [19–21], weswegen sie Menschen mit Basis-Bolus-Pentherapie angeboten werden sollten.

## Insulinpumpentherapie

Bei der Insulinpumpentherapie (oder „continuous subcutaneous insulin infusion therapy“ [CSII]) handelt es sich um eine Basis-Bolus-Therapie, bei der das Insulin subkutan mittels eines Katheters appliziert wird; kontinuierlich als sog. Basalrate (ersetzt das lang wirksame Basalinsulin[-Analogon]) und zusätzlich als Bolus (zum Essen und zur Korrektur) mittels eines schnell wirksamen Insulin(‑Analogon).

Sowohl die Vorteile der Insulinpumpentherapie (Dosierbarkeit 20-fach feiner als bei Pens, kontinuierliche Abgabe einer bis zu halbstündlich einstellbaren Basalrate, Unterstützung durch integrierten Bolusrechner) als auch die Vorteile von Glukosesensoren (kein oder nur selten notwendiges blutiges Messen, aktuelle Glukosewerte, Alarmfunktionen, Trendanzeige) prädestinieren beide Systeme für den Einsatz bei Menschen mit T1D und Personen mit anderen Diabetesformen, die eine intensive Insulintherapie, definiert als 3 oder mehr Insulininjektionen am Tag, durchführen. Insbesondere im Kindes- und Jugendalter ist die Verwendung von Insulinpumpen und CGM-Systemen als bevorzugte Therapie in allen Altersgruppen einzusetzen [22]. Aktuell verwenden in Österreich über 90 % aller Kinder und Jugendlichen mit T1D einen Glukosesensor und bis zu 80 % eine Insulinpumpentherapie. Für beides werden die Kosten von den Sozialversicherungen getragen [23].

Durch die Verwendung von Insulinpumpen und Glukosesensoren werden niedrigere HbA_1c_-Werte und eine höhere TIR erreicht, ohne dabei das Hypoglykämierisiko zu erhöhen [22].

## Sensor-unterstützte Pumpentherapie (SUP)

In der Anwendung werden CGM-Systeme oft mit Insulinpumpen im Sinne einer sensorunterstützten Insulinpumpentherapie kombiniert. Verwendung finden nach wie vor Systeme ohne Interaktion zwischen Sensor und Pumpe. Die Therapiesteuerung obliegt dabei den Anwender:innen ohne weitere digitale Hilfe. Die automatische Insulinabschaltung bei drohender Hypoglykämie – reaktiv (LGS) oder prädiktiv (PLGM) – konnte nachweislich die Häufigkeit und Dauer von Hypoglykämien reduzieren. Systeme, die ausschließlich diese Funktionen nutzten, spielen heute in der Versorgung kaum noch eine Rolle. Entsprechende Mechanismen sind jedoch weiterhin in vielen modernen AID-Systemen integriert und können aktiviert werden, wenn die Systeme nicht im AID-Modus betrieben werden [22, 24–26].

## „Automated insulin delivery“(AID)-Systeme

Diese Systeme sind derzeit die am weitesten fortgeschrittene Technologie. Bei komplexeren automatisierten Insulinpumpen/CGM-Systemen, auch als „künstliche Bauchspeicheldrüse“, „Automated insulin delivery“(AID)-Systeme bezeichnet, reguliert ein Algorithmus die Insulinzufuhr über die Pumpe unter Berücksichtigung der aktuellen, vergangenen und prädiktiven Sensor-generierten Glukoseverläufe. Im Vergleich zur Standardtherapie (Pumpe/Pen mit CGM) ist der Einsatz von AID-Systemen verbunden mit einer erhöhten TIR sowie reduzierter Hyperglykämie und Hypoglykämiehäufigkeit bei gleichzeitiger Reduktion der HbA_1c_-Werte – ein Nutzen, der sowohl in Metaanalysen randomisierter kontrollierter Studien (RCTs) als auch in retrospektiven Analysen großer Real-World-Datensätze bestätigt wurde [27, 28].

Eine Übersicht über die CE-zertifizierten Systeme in der EU gibt Tab. 1. In Österreich sind aktuell (Stand 6/2025) 2 Systeme erhältlich, bei denen die Sozialversicherungsträger die Kosten übernehmen. Das System der Firma Medtronic, 780 G (Medtronic Minimed, Inc., Northridge, CA, USA) bestehend aus der Medtronic 780 G-Pumpe, auf der auch der Algorithmus arbeitet, und dem Guardian 4-Sensor bzw. dem Simplera Sync-Sensor. Das zweite System von Ypsomed (Ypsomed, Burgdorf, Schweiz) besteht aus der YpsoPump, dem CamAPS FX-Algorithmus (CamDiab Ltd, London, UK) der als App sowohl für Android als auch für iPhones (Aplle, CA, USA) zur Verfügung steht und mit dem Glukosesensor Dexcom G6 (Dexcom, San Diego, USA) sowie dem Glukosesensor Abbott Libre 3 (Abbott, IL, USA) funktionstüchtig ist. Bei allen angeführten Systemen handelt es sich um Hybridlösungen, d. h. die basale Insulinabgabe wird durch den Algorithmus automatisch moduliert, der Mahlzeitenbolus muss weiterhin manuell abgegeben werden.

**Tab. 1:** CE-zertifizierte AID-Systeme. (Adaptiert nach Biester et al. [29])

	CamAPS FX	Diabeloop DBLG1/bDBL4T	Omnipod 5	MiniMed 780 G	T‑Slim X: 2 mit Control IQ
Insulinpumpe	Dana R/S, Dana‑i od. mylife YpsoPump	Accu-Chek Insight/Kaleido-Pumpe	Omnipod 5 ACE-Pump (Pod)	MiniMed 780 G	T‑Slim X: 2
Glukosensor	Dexcom G6, Libre 3	Dexcom G6	Dexcom G6/G7, Libre 2 plus	Guardian 4 Simplera Sync	Dexcom G6/G7, Libre 2 plus
Sensorfunktionsdauer	10 Tage (Dexcom G6) bzw. 15 Tage (Libre 3)	10 Tage	10 Tage (Dexcom G6/G7) bzw. 15 Tage (Libre 2 plus)	7 Tage	10 Tage (Dexcom G6/G7) bzw. 15 Tage (Libre 2 plus)
Nötige Blutzuckerkontrollen	Keine	Keine	Keine	Bei Start des SG-Modus nötig	Keine
Art des Algorithmus	MPC	MPC	MPC	PID mit Fuzzy Logic und MPC-Anteil	MPC
Plattform des Algorithmus	Android/iOS-Smartphone	Handgerät	Handgerät	In der Pumpe	In der Pumpe
Altersbeschränkung	≥ 1 Jahr (auch für Schwangere)	12 bis 18 Jahre (DBL4T) > 18 Jahre (DBLG1)	≥ 2 Jahre	≥ 7 Jahre	≥ 6 Jahre
Glukoseziel (mg/dl)	80–200	100–180	110–150 mg/Tag (in 10er-Schritten)	100, 110 oder 120	110–150 (in 10er-Schritten)
Automatische Korrekturbolusgabe	Nein	Ja	Nein	Ja	Ja
Möglichkeit der Datenverfügbarkeit	Automatisch, Glooko	Download, Glooko (Sensordaten Clarity)	Download, z. B. Glooko	Automatisch, CareLink	Download, Glooko (Sensordaten Clarity)
Sonstiges	„Boost-Modus“	Variable „Aggressivität“	–	Per Handy anzusehen	Nachtmodus
Temporäres Ziel erhöhen	„Ease off“/Aktivitätsmodus	Zen-Modus (20–40 mg/dl höher als aktuelles Ziel)	Temp. Ziel (150 mg/dl)	Temp. Ziel (150 mg/dl)	Aktivitätsmodus
Verfügbar in Österreich (06/2025)	Ja (mit mylife YpsoPump)	Nein	Nein	Ja	Nein

*PID* „proportional integral derivative“, *MPC* „model predictive control“

Unterschiede bestehen in den Komponenten: Je nach System kann es sein, dass trotz eines Glukosesensors zusätzlich zur Kalibrierung auch einzelne präprandiale kapilläre Glukosewerte nötig sind. Andere Sensoren kommen laut Herstellerangabe ohne kapilläre Glukosewerte aus. Manche Systeme können Glukosewerte per Bluetooth-Verbindung auf ein Smartphone übermitteln. Es gibt auch Systeme, bei denen der Steuerungsalgorithmus nicht in der Pumpe eingebaut ist, sondern sich auf einem externen Gerät bzw. als App auf einem Mobiltelefon befindet (s. Tab. 1).

Aufgrund von unterschiedlichsten Barrieren in der AID-Verfügbarkeit hat sich international die Nutzung von Open-source-AID-Systemen entwickelt. Open-source-Technologie zeichnet sich durch global freie Verfügbarkeit von Codes im Internet aus, durchläuft kein offizielles Zulassungsverfahren, und die Verwendung erfolgt daher auf eigene Verantwortung der Anwender:innen; ein Positionspapier der ÖDG geht näher darauf ein [30].

## Einsatz von Diabetestechnologie in der Schwangerschaft

Einige RCTs haben demonstriert, dass die Verwendung von CGM während der Schwangerschaft die glykämische Kontrolle und das neonatale Outcome verbessern [31, 32], daher wird der Einsatz von CGM in der Schwangerschaft von Frauen mit Typ-1-Diabetes unbedingt empfohlen. In einer sekundären Analyse der CONCEPTT-Studie hatten Kinder von Pumpenuserinnen im Vergleich zu MDI-Userinnen eine höhere Wahrscheinlichkeit, auf einer Intensivstation aufgenommen zu werden und eine neonatale Hypoglykämie, welche mit Glukose behandelt werden muss, zu erleiden. Die Lebensqualität wurde dagegen als besser beschrieben. Es zeigte sich im ersten Trimester kein HbA_1c_-Unterschied zwischen den Gruppen, wohl aber in der 34. SSW. Entscheidend für dieses Ergebnis war, dass Pumpenuserinnen in der 24. SSW um 5 % weniger TIR (63–140 mg/dl) verbrachten als MDI-Userinnen trotz vergleichbarer Ergebnisse im ersten und dritten Schwangerschaftsdrittel [33]. Eine weitere Analyse fand keinen Unterschied im Essverhalten zu den MDI-Userinnen. Es blieb die Tatsache, dass die Insulindosis nicht adäquat angepasst wurde [33] und dass die unterschiedliche Kinetik des schnell wirksamen Insulins in der Schwangerschaft berücksichtigt werden muss. Vieles spricht in diesem Zusammenhang dafür, dass eine Automatisierung der Insulindosis sich günstig auswirken kann. So konnte in der RCT-AiDAPT unter Verwendung des CamAPS FX-Algorithmus die TIR bei Müttern mit Typ-1-Diabetes signifikant ohne Sicherheitsprobleme verbessert werden [34]. Die Ergebnisse haben zu einer Zulassung des Systems in der Schwangerschaft geführt. In der RCT-CRISTAL mit der Minimed 780 G (PID-Algorithmus) konnte keine Verbesserung der TIR erreicht werden. Die nächtliche Zeit im Zielbereich war höher, und die Frauen berichteten über eine höhere Behandlungszufriedenheit [35]. Inwieweit die beiden AID-Systeme nachteilige Auswirkungen auf die Gesundheit von Müttern und Neugeborenen verhindern, konnte durch die Studien nicht unmittelbar beantwortet werden.

Eine große Kohortenstudie berichtet über eine signifikante Verbesserung der täglichen Glukoseprofile, geringere Glukosevariabilität und geringere durchschnittliche Glukosewerte mit CGM verglichen mit der Blutglukosemessung bei Frauen mit Schwangerschaftsdiabetes. Hohe durchschnittliche Glukosewerte waren signifikant mit hohem Geburtsgewicht assoziiert und waren ein unabhängiger Risikofaktor für Präeklampsie und neonatales Outcome [36]. Rezentere Studien konnten keinen eindeutigen Vorteil von CGM zeigen, wobei in den Studien nicht zwischen Insulin-behandelten und nicht Insulin-behandelten Frauen unterschieden wurde [37, 38].

## Einsatz von Technologie im Kindes- und Jugendalter

Die Insulinpumpentherapie, die Verwendung von Glukosesensoren und idealerweise die Anwendung von automatisierten Insulinabgabesysteme (AIDs) soll allen Kindern und Jugendlichen mit Typ-1-Diabetes jeglichen Alters von Beginn an empfohlen und ermöglicht werden [20, 21].

Alle derzeit erhältlichen und im Kindesalter zugelassenen Systeme (ab 1. Lebensjahr CamAPS FX, ab 2. Lebensjahr Medtronic 780 G) sind AID-Systeme, bei denen die Mahlzeitenboli manuell abgegeben werden müssen. Die Anwendung dieser Systeme geht mit einer Steigerung der Zeit im Zielbereich und einer Reduktion der HbA_1c_-Werte und der Hypoglykämiehäufigkeit einher [39, 40].

Eine gute metabolische Einstellung von Beginn an ist prognostisch essenziell [20, 21, 41], daher ist es notwendig, neben der altersgerechten Diabetesschulung eine individualisierte, altersadäquate Therapie anzuwenden, um eine hohe Therapiezufriedenheit und Compliance zu erreichen. Hautreaktionen bei der Nutzung von CGM-Sensoren und Kathetern sind häufig und können von einfachen Hautirritationen bis hin zu allergischen Kontaktekzemen reichen. Diese können sowohl durch mechanische Reibung als auch durch Klebstoffe und Materialien der Sensoren verursacht werden. Regelmäßige Kontrollen der Katheter- und Sensorstellen sollen Teil der Follow-up-Untersuchungen sein. Eine sorgfältige Hautpflege, der regelmäßige Wechsel der Setzstellen sowie der Einsatz hautschonender Produkte und Barrierefilme sind wichtige Maßnahmen, um Hautirritationen vorzubeugen [42]. Dies gilt für alle Menschen mit Diabetes, welche diese Systeme nutzen. Ein möglichst früher Einsatz von AID-Systemen, idealerweise schon wenige Tage bis Wochen nach Diagnosestellung, wird empfohlen, da er die glykämische Kontrolle langfristig – bis zu 48 Monate – deutlich verbessert im Vergleich zu Systemen ohne Automatisierung [43–45].

## Einsatz von Diabetestechnologie bei Menschen mit Diabetes mellitus Typ 2

Obwohl in großen klinischen Studien mit isCGM nur minimale HbA_1c_-Verbesserungen in Populationen mit T2D auch mit einer intensiven Insulintherapie erreicht werden konnten, war die Anwendung von isCGM mit einer signifikanten Reduktion von Hypoglykämien sowie einer verbesserten Lebensqualität vergesellschaftet [46–48]. Üblicherweise wird die Insulintherapie aber mit einer basal unterstützten oralen Therapie (BOT) begonnen. Dabei konnte eine RCT eine signifikante HbA_1c_-Reduktion unter Einsatz von CGM zeigen [49]. Retrospektiv konnte sowohl bei Personen mit T2D mit BOT aber auch bei Personen ohne Insulintherapie eine Verringerung der akuten diabetesbezogenen Ereignisse und Hospitalisierungen belegt werden [62, 63]. Eine verbesserte glykämische Kontrolle, eine verbesserte Gewichtskontrolle wie eine Veränderung des Verhaltens konnten in einer limitierten Anzahl von Studien von großer Heterogenität für die Anwendung von CGM bei Menschen, die keine intensive Insulintherapie durchführen, dargelegt werden [50, 51].

Die Insulinpumpentherapie kann bei Menschen mit T2D die HbA_1c_-Werte signifikant reduzieren, wobei v. a. jene mit der schlechtesten glykämischen Kontrolle und den höchsten Insulindosen profitieren. Bei einem HbA_1c_ < 8 % (64 mmol/mol) ist der Effekt gering, wenn auch die Menschen persönlich zufrieden sind [52]. Seit dem Abschluss der OpT2mise-Studie, einer RCT für Insulinpumpentherapie bei Menschen mit T2D, sind viele neue medikamentöse Möglichkeiten entwickelt worden. Direkte Vergleiche oder Kombinationen von Pumpentherapie mit neuen Medikamenten finden sich nicht in der Literatur. Zuletzt zeigte eine 13-wöchige RCT, an welcher Personen mit insulinbehandeltem T2D teilnahmen, dass die Verwendung von AID mit einer stärkeren Senkung der HbA_1c_-Werte verbunden war als mit CGM allein [53].

## Einsatz von Diabetestechnologie bei Menschen mit „anderen spezifischen Diabetesformen“

Die unter der Kategorie „andere spezifische Diabetesformen“ zusammengefassten Erkrankungen stellen pathophysiologisch und therapeutisch eine sehr heterogene Krankheitsgruppe dar. Die genauere Beschreibung der einzelnen Formen sowie deren Therapiemöglichkeiten werden in den ÖDG-Diabetesleitlinien an anderer Stelle beschrieben.

Grundsätzlich gibt es nur eine geringe Anzahl an wissenschaftlich relevanten Arbeiten, die den Einsatz von Technologien auch bei den häufigeren Formen, wie z. B. dem pankreopriven Diabetes, bisher untersucht haben. Es ist aber davon auszugehen, dass insbesondere absolut insulinabhängige Diabetesformen, wie z. B. Menschen nach totaler Pankreatektomie, aufgrund ihrer Ähnlichkeit zu T1D und der instabilen Stoffwechsellage mit einer deutlich erhöhten Neigung zu Hypoglykämien besonders von der Nutzung der neuen Diabetestechnologien (CGM- und AID-Systemen) profitieren [54]. Sofern Menschen mit Diabetes die zur Nutzung dieser Technologien notwendigen Voraussetzungen erfüllen, soll der Einsatz dieser aus oben genannten Gründen großzügig erfolgen.

Für den Posttransplantationsdiabetestyp gibt es eine Studie mit geringer Fallzahl, die einen Vorteil für den Einsatz der Insulinpumpentherapie bei nierentransplantierten Patient:innen im Vergleich zur Injektionstherapie zeigt [55].

Für den Diabetes im Rahmen einer zystischen Fibrose konnte in einem Review trotz des Vorliegens von zumindest 14 Studien keine ausreichende Evidenz für die Nutzung von CGM-Systemen nachgewiesen werden. Laut Autoren gibt es aber auch keine potenziellen negativen Effekte beim Einsatz dieser Technologien [56–58].

## Technologie als Grundlage für telemedizinische Betreuung

Alle Insulinpumpen, CGM-Systeme und AID-Systeme können über cloudbasierte Software ausgelesen werden bzw. werden automatisch in die entsprechende Cloud hochgeladen. Damit besteht die ideale technische Grundlage für eine telemedizinische Betreuung, die v. a. während der SARS-CoV-2-Pandemie in vielen Diabeteszentren zu einer neuen Realität geworden ist [59, 60]. Auch über die Pandemie hinaus birgt die Telemedizin großes Potenzial in der Langzeitbetreuung von Menschen mit Diabetes mellitus. Um Telemedizin in die Versorgungsstruktur implementieren zu können, bedarf es allerdings einer soliden Planung und Umsetzung unter Berücksichtigung rechtlicher und datenschutzrechtlicher Grundlagen [61].

## Vermittlung von Theorie und Praxis

Die Implementierung und Verwendung von Diabetestechnologie muss fundiert vermittelt und trainiert werden [62]. Strukturierte formale Schulungsprogramme haben sich als effektiv im Sinne von verbesserter glykämischer Kontrolle, Akzeptanz und Zufriedenheit der Anwender:innen erwiesen [63, 64]. Vor allem die standardisierte CGM-Analyse mittels AGP (ambulantes Glukoseprofil) sollte von medizinischen Fachkräften im Diabetesbereich beherrscht und die Daten sollten in Analogie zu Laborparametern (z. B. HbA_1c_) zur Verlaufskontrolle dokumentiert werden. Das multidisziplinäre Schulungsteam sollte (pädiatrische) Diabetolog:innen, Diabetesberater:innen, Diätolog:innen, Psycholog:innen sowie Sozialarbeiter:innen umfassen. Um eine qualitativ hochwertige Versorgung zu gewährleisten sind kontinuierliche Fortbildungen der Diabetesteams unverzichtbarer Teil eines erfolgreichen Qualitätsmanagements.

### Therapieempfehlung der ÖDG für den Einsatz von Diabetestechnologie (CSII; CGM; HCL, inklusive Option zur Telemedizin bei Menschen mit Diabetes mellitus)

#### Evidenzklassen (EK)

Ia: systematische Übersichtsarbeiten von Studien der Evidenzstufe Ib.

Ib: randomisierte vergleichende klinische Studien.

IIa: systematische Übersichtsarbeiten von Studien der Evidenzstufe IIb.

IIb: prospektive, insbesondere vergleichende Kohortenstudien.

III: retrospektive Studien.

IV: Evidenz außerhalb von Studien (Meinungen anerkannter Experten, Assoziationsbeobachtungen, pathophysiologische Überlegungen oder deskriptive Darstellungen, Berichte von Expertenkomitees, Konsensuskonferenzen, Einzelfallberichte).

1. Der Routineeinsatz von CGM wird empfohlen bei
  allen Menschen mit Diabetes mellitus, die eine intensive Insulintherapie, definiert als 3 oder mehr Insulininjektionen am Tag, durchführen oder eine Insulinpumpe nutzen (Ia), sowie allen Menschen mit Diabetes bei jeder Art von Insulintherapie (Ib),
  neu diagnostiziertem Diabetes mellitus Typ 1 (IIa)
  allen Menschen mit Hypoglykämien (häufige/schwere Hypoglykämien, nächtliche Hypoglykämien, Hypoglykämiewahrnehmungsstörung) (Ia),
  allen schwangeren Frauen mit Diabetes mellitus, die eine intensive Insulintherapie durchführen unabhängig vom Diabetestyp (Ia), sowie Frauen mit Schwangerschaftsdiabetes, die eine Insulintherapie durchführen (Ib).
2. Der Routineeinsatz von CGM kann empfohlen werden bei
  Menschen mit T2D, um individuelle glykämische Ziele zu erreichen und zu halten (IIb),
  Frauen mit Schwangerschaftsdiabetes, welche keine Insulintherapie durchführen (IIb).
3. Der diagnostische und intermittierende Einsatz von CGM zur Therapieentscheidung und Therapiekontrolle wird empfohlen bei
  neu diagnostiziertem Diabetes mellitus (IV)
  Menschen, mit problematischen Hypoglykämien, die CGM aber nicht in der Routine nutzen (IV),
  Menschen mit Typ-2-Diabetes ohne Insulin, welche CGM als Schulungstool episodisch/intermittierend nutzen (IV),
  Menschen, die Medikamente verabreicht bekommen, die eine Hyperglykämie zur Folge haben können (IV).
4. Insulinpumpentherapie ohne CGM soll allen Menschen mit intensiver Insulintherapie zur Verfügung stehen, die aufgrund von Hautproblemen oder psychischer Überforderung kein CGM nutzen können. Dabei soll der Blutzucker zumindest 4‑mal täglich kontrolliert werden (Ib).
5. Die Insulinpumpentherapie kann bei Menschen mit Typ-2-Diabetes mit schlechter glykämischer Kontrolle und hohen Insulindosen eingesetzt werden (Ia).
6. Insulinpumpentherapie mit CGM oder SAP wird allen Menschen mit Diabetes, die eine intensive Insulintherapie durchführen, empfohlen, die sich aktiv dafür entscheiden oder ihre Therapieziele mit einer MDI nicht erreichen (Ia).
7. Insulinpumpentherapie mit PLGS wird Menschen mit Diabetes mit Hypoglykämien empfohlen (Ib).
8. „Automated insulin delivery“(AID)-Systeme werden allen Menschen mit Typ-1-Diabetes empfohlen, um ihre TIR zu erhöhen, Hyper- und Hypoglykämien zu verhindern und um ihre Lebensqualität zu erhöhen. Sie sind bevorzugt einzusetzen (Ia).
9. „Connected insulin pens“ sollen Menschen mit CGM und Pentherapie empfohlen werden, um das Injektionsverhalten besser verstehen und adaptieren zu können (IIa).

## Interessenkonflikt

I. Schütz-Fuhrmann erhielt Vortrags- und Beratungshonorare von Abbott Diabetes Care, Medtronic, Dexcom, Insulet, B. Rami-Merhar hat in den letzten 36 Monaten Vertragshonorare für Vorträge oder Advisory Boards erhalten: Eli Lilly, Medtronic, Insulet, Abbott, Sanofi, S.E. Hofer hat in den letzten 36 Monaten von folgenden Unternehmen, die auch fördernde Mitglieder der ÖDG sind, Forschungsunterstützungen und/oder Honorare erhalten: Eli Lilly, Sanofi und Ypsomed, E. Fröhlich-Reiterer hat in den letzten 36 Monaten von folgenden Firmen Honorare/Forschungsgelder erhalten: Eli Lilly, Medtronic, Novo Nordisk, Roche, M. Tauschmann hat in den letzten 36 Monaten von folgenden Firmen Vortrags- bzw. Beratungshonorare erhalten: Abbott Diabetes Care, Eli Lilly, Medtronic, J.K. Mader erhielt Vortragshonorare von AstraZeneca, Abbott Diabetes Care, Böhringer Ingelheim, Eli Lilly, Medtronic Minimed, Menarini, Merck Sharp & Dohme, Nintamed, Novo Nordisk, Roche Diabetes Care, Sanofi-Aventis, Servier, Wellion, Ypsomed. Advisory Board: Abbott Diabetes Care, Böhringer Ingelheim, Eli Lilly, Medtronic Minimed, Novo Nordisk, Roche Diabetes Care, Sanofi-Aventis. Gesellschafterin decide Clinical Software GmbH. Forschungsgelder von A. Menarini Diagnostics, B. Braun, Metronom Health, Novo Nordisk, Profusa Inc., Roche Diabetes Care, Sanofi-Aventis., M. Resl hat Vortragshonorare und Honorare für Beratungstätigkeit von folgenden Firmen erhalten: Boehringer Ingelheim, Sanofi-Aventis, Novo Nordisk, Dexcom, A. Kautzky-Willer hat von folgenden Unternehmen, die auch fördernde Mitglieder der ÖDG sind, Forschungsunterstützungen und/oder Honorare erhalten: Amgen, Boehringer Ingelheim, El Lilly, Merck Sharp & Dohme, Novartis, Novo Nordisk, Roche, Y. Winhofer-Stöckl hat von folgenden Unternehmen, die auch fördernde Mitglieder der ÖDG sind, Forschungsunterstützungen und/oder Honorare erhalten: Abbott, AstraZeneca, Amgen, Boehringer Ingelheim, Eli Lilly, Merck Sharp & Dohme, Medtronic, Novartis, Novo Nordisk, Roche, Sanofi-Aventis, T. Wascher hat von folgenden Unternehmen Honorare erhalten: Abbott, Dexcom, Eli Lilly, Novo Nordisk, L. Stechemesser hat von folgenden Unternehmen, die auch fördernde Mitglieder der ÖDG sind, Forschungsunterstützungen und/oder Honorare erhalten: Abbott, Roche, Medtronic, S. Zlamal-Fortunat hat in den letzten 36 Monaten Honorare für Vorträge und Advisory Boards von Novo Nordisk, Eli Lilly, AstraZeneca, Boehringer Ingelheim, Medtronic, MSD, Daiichi Sankyo und Novartis erhalten, und R. Weitgasser erhielt Vortrags- und Beratungshonorare von Abbott Diabetes Care, Eli Lilly, Medtrust, Novo Nordisk, Roche Diabetes Care, Sanofi. J. Harreiter gibt an, dass kein Interessenkonflikt besteht.
